# Quantifying degree of foot use impairment in hemiplegic gait using center-of-pressure trajectory vector difference integrals

**DOI:** 10.1186/1757-1146-7-S1-A64

**Published:** 2014-04-08

**Authors:** TC Pataky, H Tanaka, M Hashimoto

**Affiliations:** 1 Department of Bioengineering, Shinshu University, Ueda, Nagano, Japan

## Introduction

Gait impairment manifestations can vary greatly amongst hemiplegic patients, so it is difficult to derive a single parameter to summarize impairment severity. In a separate study we found that the variance-normalized integrated difference amongst center-of-pressure (COP) trajectories was positively correlated with gait impairment severity. The purpose of this study was to test whether this COP trajectory difference parameter could also differentiate involved from uninvolved limbs in hemiplegia.

## Methods

In-shoe COP trajectories were collected from (a) eight healthy students during treadmill walking, and (b) six hemiplegic patients during level-ground walking. Ten time-normalized COP trajectories per subject were analyzed. Each subject was compared to the mean Control trajectory by first computing the Hotelling’s *T*^2^ statistic at each point in time (Eqn.1), then integrating over stance phase (Eqn.2):(1)(2)

**Figure 1:**
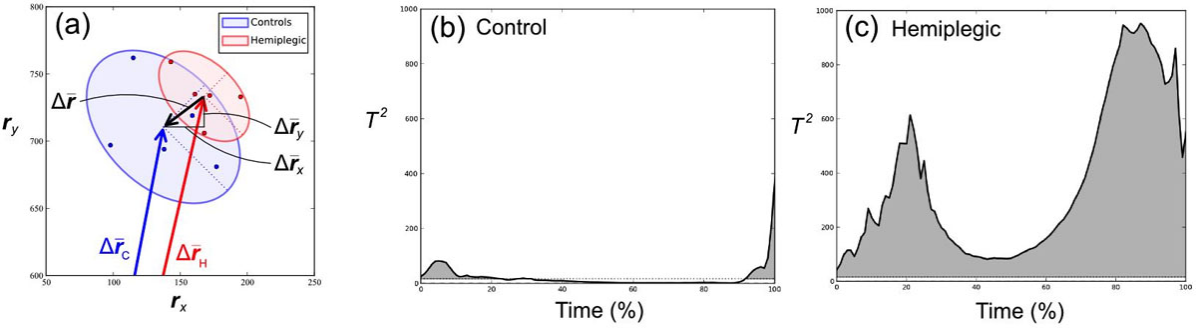
(a) Depiction of instantaneous vector difference with covariance ellipses. (b) Example Control *T*^2^ trajectory depicting COP trajectory differences in time. (c) Example Hemiplegic *T*^2^ trajectory.

Here *n*=10 is the number of footsteps,  is the instantaneous position difference between a subject’s mean COP and the mean Control COP, and ***W*** is its covariance (Figure 1a).

## Results

Control subjects’ COP trajectories were qualitatively more similar to the Control mean than were those of Hemiplegic subjects. Compared to Controls (9.4 ± 4.3) (Figure 1b), hemiplegic subjects exhibited greater *T*^2^ integrals in both the involved (123.7 ± 117.3) (*p*=0.016) and uninvolved limbs (30.2 ± 14.4) (*p*=0.002) (Figure 1c). The *T*^2^ integral also tended to be greater in the involved vs. uninvolved limbs within-subjects (+252.2% ± 200.0%) (*p*=0.048).

## Discussion

These results suggest that the *T*^2^ integral appears to be useful metric for summarizing stance-phase foot use differences both within- and between-subjects. A broader range of clinical conditions are currently under investigation.

